# Atrial Fibrillation Is Associated with Increased Mortality in Patients Presenting with Ventricular Tachyarrhythmias

**DOI:** 10.1038/s41598-019-49325-4

**Published:** 2019-10-03

**Authors:** Michael Behnes, Jonas Rusnak, Gabriel Taton, Tobias Schupp, Linda Reiser, Armin Bollow, Thomas Reichelt, Niko Engelke, Dominik Ellguth, Philipp Kuche, Ibrahim El-Battrawy, Siegfried Lang, Christoph A. Nienaber, Kambis Mashayekhi, Muharrem Akin, Thomas Bertsch, Dennis Ferdinand, Christel Weiss, Martin Borggrefe, Ibrahim Akin

**Affiliations:** 1 First Department of Medicine, University Medical Centre Mannheim (UMM), Faculty of Medicine Mannheim, University of Heidelberg, European Center for AngioScience (ECAS), and DZHK (German Center for Cardiovascular Research) partner site Heidelberg/Mannheim, Mannheim, Germany; 2 Royal Brompton and Harefield Hospitals, NHS, London, United Kingdom; 3 Department of Cardiology and Angiology II, University Heart Center Freiburg, Bad Krozingen, Germany; 4 Hannover Medical School, Department of Cardiology and Angiology, Hannover, Germany; 5 Institute of Clinical Chemistry, Laboratory Medicine and Transfusion Medicine, General Hospital Nuremberg, Paracelsus Medical University, Nuremberg, Germany; 6 Institute of Biomathematics and Medical Statistics, University Medical Center Mannheim (UMM), Faculty of Medicine Mannheim, Heidelberg University, Mannheim, Germany

**Keywords:** Atrial fibrillation, Ventricular fibrillation

## Abstract

Heterogenous data about the prognostic impact of atrial fibrillation (AF) in patients with ventricular tachyarrhythmias exist. Therefore, this study evaluates this impact of AF in patients presenting with ventricular tachyarrhythmias. 1,993 consecutive patients presenting with ventricular tachyarrhythmias (i.e. ventricular tachycardia and fibrillation (VT, VF)) on admission at one institution were included (from 2002 until 2016). All medical data of index and follow-up hospitalizations were collected during the complete follow-up period for each patient. Statistics comprised univariable Kaplan-Meier and multivariable Cox regression analyses in the unmatched consecutive cohort and after propensity-score matching for harmonization. The primary prognostic endpoint was long-term all-cause mortality at 2.5 years. AF was present in 31% of patients presenting with index ventricular tachyarrhythmias on admission (70% paroxysmal, 9% persistent, 21% permanent). VT was more common (67% versus 59%; p = 0.001) than VF (33% versus 41%; p = 0.001) in AF compared to non-AF patients. Long-term all-cause mortality at 2.5 years occurred more often in AF compared to non-AF patients (mortality rates 40% versus 24%, log rank p = 0.001; HR = 1.825; 95% CI 1.548–2.153; p = 0.001), which may be attributed to higher rates of all-cause mortality at 30 days, in-hospital mortality and mortality after discharge (p < 0.05) (secondary endpoints). Mortality differences were observed irrespective of index ventricular tachyarrhythmia (VT or VF), LV dysfunction or presence of an ICD. In conclusion, this study identifies AF as an independent predictor of death in patients presenting consecutively with ventricular tachyarrhythmias.

## Introduction

Atrial fibrillation (AF) represents the most common arrhythmia worldwide, since about 33.5 million individuals were estimated to suffer from AF in 2010^1^. AF is associated with increased comorbidity, such as stroke or heart failure, a 2-fold increase of mortality in both men and women and around 3% in anticoagulated AF patients^1,2^. In minor part, death may rarely be attributed to stroke, whereas death from progressive heart failure and sudden cardiac death (SCD) is more common in AF patients^2^.

SCD and ventricular tachyarrhythmias are predominantly seen in the presence of coronary artery disease (CAD) and acute myocardial infarction (AMI). Accordingly, about half of cardiac deaths after AMI are related to ventricular tachyarrhythmias, such as ventricular tachycardia (VT) or fibrillation (VF) and consecutive SCD^3–6^. Accumulating evidence suggests a mechanistic link in between AF and ventricular tachyarrhythmias, which may be explained by reduced ventricular refractoriness and pro-arrhythmic short-long-short sequences preceding the onset of ventricular tachyarrhythmias in the presence of AF rather than in sinus rhythm^7^.

According to the literature, several community-based studies demonstrated a higher incidence of future SCD in AF patients at long-term follow-up^7–10^. A sub-analysis of the Engage AF-TIMI 48 trial showed a rate of SCD estimated at 45% of cardiovascular deaths in pre-selected AF patients being investigated initially for the effectiveness of edoxaban compared to warfarin for stroke prevention^9^. Furthermore, the Oregon-SUD study found a higher rate of AF related to SCD in 652 SCD patients compared to age- and sex- matched CAD controls^11^. Notably the increasing SCD risk was no longer attributed to AF in the presence of heart failure^11,12^.

However, no data is currently available, whether the presence of AF may be associated independently with mortality in consecutive real-life patients presenting on admission with ventricular tachyarrhythmias. Therefore, this study evaluates the differences of short- and long-term survival of patients surviving ventricular tachyarrhythmias on admission depending on the presence or absence of AF.

## Methods

### Study patients

The present study is derived from the “Registry of malignant arrhythmias and sudden cardiac death” (RACE-IT), which included retrospectively all patients presenting with at least one episode of ventricular tachyarrhythmias and sudden cardiac arrest between 2002 and 2016 at one institution, as recently been outlined^13^. Ventricular tachyarrhythmias included ventricular tachycardia (VT) and fibrillation (VF) as defined by current European guidelines^14^. Sustained VT was defined by duration of more than 30 seconds or by causing hemodynamic collapse. Non-sustained VT was defined by duration of less than 30 seconds both with wide QRS complex (≥120 milliseconds) at a rate greater than 100 beats per minute^14^. Ventricular tachyarrhythmias were documented by 12-lead electrocardiogram (ECG), ECG tele- monitoring, ICD or by external defibrillator monitoring. Documented VF was treated by external defibrillation and in case of prolonged instability with additional intravenous anti-arrhythmic drugs during cardiopulmonary resuscitation (CPR).

This study is based on a retrospective data analysis/registry and has been approved by the local ethics commission II of the faculty of Medicine Mannheim, University of Heidelberg, where no informed consent was deemed necessary for this study (ethical approval number 2016612NMA) (ClinicalTrials.gov identifier: NCT02982473). All methods were carried out in accordance with relevant guidelines and regulations.

### Definition of study groups, inclusion and exclusion criteria

For the present analysis only patients presenting with and surviving ventricular tachyarrhythmias at index hospital stay were included. Risk-stratification was performed according to the presence of AF according to European guidelines^2^. Documentation of AF was derived from ECG recording on admission and medical history being documented within the electronic hospital information system. Paroxysmal AF was defined as self-terminating in most cases within 48 hours and lately for up to 7 days, including AF episodes that are cardioverted within 7 days. Persistent AF lasts longer than 7 days including episodes terminated by cardioversion either by drugs or by direct current cardioversion after 7 days or more. Permanent AF was defined as accepted by the patient and physician without pursuing further rhythm control.

Patients with early cardiac death defined as occurring <24 hours after onset of ventricular tachyarrhythmias or an assumed unstable cardiac condition on index admission were excluded from the present study^14^. Each patient was counted only once for inclusion when presenting with the first episode of ventricular tachyarrhythmias.

### Study endpoints

The primary endpoint was defined as long-term all-cause mortality at 2.5 years of follow-up. Secondary endpoints were all-cause mortality at 30-days, in-hospital death at index and all-cause mortality of surviving patients of index hospitalization (i.e. after discharge). Risk stratification was performed within subgroups of VT, VT, and AF subtypes, LV dysfunction and overall implantable cardioverter defibrillators (ICD).

All-cause mortality was documented using our electronic hospital information system and by directly contacting state resident registration offices (“bureau of mortality statistics”) across Germany. Identification of patients was verified by place of name, surname, day of birth and registered living address. In 48 patients, no data on patients’ survival could have been retrieved, as those patients were even not reachable by telephone, and therefore these patients were excluded from final analyses (corresponding lost to follow-up rate of 1.7%).

### Statistical methods

The following analyses were applied stepwise to evaluate the prognostic impact of predefined variables for all-cause mortality: Firstly, within the entire cohort, uni-variable stratification was performed using the Kaplan-Meier method with comparisons between groups using uni-variable hazard ratios (HR) given together with 95% confidence intervals. Secondly, propensity score analyses were performed, since this study includes consecutively all patients with ventricular tachyarrhythmias without randomization^15,16^. Accordingly, a propensity score (probability for belonging to AF = yes) was calculated for each individual based on the same predefined variables (see below). Afterwards, matched pairs were created using the method of nearest neighbor matching with a caliper distance of 5%. This means: each pair consisted of one individual with AF = yes and one individual with AF = no, respectively, whose propensity scores differed by less than 5%. We found 496 pairs with mean propensity score 0.3722 +/− 0.1412 (AF = 0) and 0.4134 +/− 0.1379 (AF = 1). Uni-variable stratification was re-calculated according to Kaplan-Meier methods for each above-said subgroup within the propensity-matched cohort. Thirdly, multivariable Cox regression models were developed using the “forward selection” option, where only statistically significant variables (p < 0.05) were included and analyzed simultaneously. Multivariable Cox regressions were applied within the entire and propensity-matched cohorts.

Predefined variables being used for propensity score matching (step 2) and multivariable Cox-regressions (step 3) included: baseline parameters (age, gender), chronic diseases (diabetes, chronic kidney disease (glomerular filtration rate < 90 mL/min/1.73 m^2^)), acute comorbidities (acute myocardial infarction, ST segment elevation myocardical infarction (STEMI), Non ST segment elevation myocardical infarction (NSTEMI), left ventricular ejection fraction (LVEF) <35%, cardiogenic shock, cardiopulmonary resuscitation (CPR), presence of an ICD, presence of a shockable rhythm (i.e. VT/VF) at index, and presence of AF.

Long-term follow-up period for evaluation of the primary endpoint was set at the median survival of AF patients to guarantee complete survival of at least 50% of affected patients. Patients not meeting long-term follow-up were censored.

The result of a statistical test was considered as a statistical trend for p < 0.1 and significant for p < 0.05 and SAS, release 9.4 (SAS Institute Inc., Cary, NC, USA) was used for statistics.

Quantitative data are given as mean ± standard error of mean (SEM), median and interquartile range (IQR), and ranges depending on the distribution of the data and were compared using the Student’s *t* test for normally distributed data or the Mann-Whitney *U* test for nonparametric data. Deviations from a Gaussian distribution were tested by the Kolmogorov-Smirnov test. Spearman’s rank correlation for nonparametric data was used to test univariate correlations. Qualitative data are presented as absolute and relative frequencies and compared using the Chi² test or the Fisher’s exact test, as appropriate. Additionally, standardized mean differences (d) were applied in addition to p values for the comparisons of patient’s characteristics between females and males. (d) was assessed calculated with a logit model. Values of (d) < 0.2 were defined similarity between groups, whereas (d) > 0.2 defined relevant differences in patients’ characteristics between AF and non-AF patients^17^.

## Results

### Entire unmatched study cohort

Within the unmatched study population of 1,993 consecutive patients presenting with ventricular tachyarrhythmias on admission at our institution, a history of AF was present in 31% of patients. Most patients suffered from paroxysmal (70%), followed by permanent (21%) and persistent AF (9%). The rate of VT was significantly higher in AF patients (67% versus 59%; p = 0.001), whereas VF was more common in non-AF patients (41% versus 33%; p = 0.001) (Table 1, left columns). AF patients had a higher cardiovascular risk profile and a higher rate of prior heart failure, CAD, valvular heart disease, ICD, chronic kidney disease and obstructive pulmonary disease. At index presentation, rates of acute myocardial infarction, cardiogenic shock, non-ischemic cardiomyopathy, CAD and CPR were equally distributed in both groups. Non-AF patients underwent PCI more often at index mostly at the LAD. AF patients presented with higher rates of LVEF <35% alongside higher rates of overall ICD, beta-blockers, digitalis, amiodarone and anticoagulant therapies (Table 1, left columns).

**Table 1:** Patients’ characteristics.

Characteristic	Before matching (n = 1,993)					After matching (n = 992)					
	non-AF (n = 1,376; 69%)		AF(n = 617; 31%)		p value	non-AF (n = 496; 50%)		AF (n = 496; 50%)		p value	(d)
**Gender**, n (%)											
Male	995	(72)	461	(75)	0.263	387	(78)	387	(76)	0.496	0.043
**Age**, median (range)	63 (14–92)		72 (23–97)		**0**.**001**	70 (16–92)		72 (23–94)		**0**.**034**	0.053
**Ventricular tachyarrhythmias**, n (%)											
Ventricular tachycardia	814	(59)	416	(67)	**0**.**001**	319	(64)	333	(67)	0.349	0.060
Ventricular fibrillation	562	(41)	201	(33)	**0**.**001**	177	(36)	163	(33)	0.349	0.060
**Type of atrial fibrillation** n (%)											
Paroxysmal	0	(0)	434	(70)	**0**.**001**	0	(0)	343	(69)	**0**.**001**	
Persistent	0	(0)	55	(9)		0	(0)	44	(9)		0.210
Permanent	0	(0)	128	(21)		0	(0)	109	(22)		
**Cardiovascular risk factors**, n (%)											
Arterial hypertension	728	(53)	431	(70)	**0**.**001**	323	(65)	347	(70)	0.104	0.103
Diabetes mellitus	323	(23)	188	(30)	**0**.**001**	150	(30)	147	(30)	0.835	0.013
Hyperlipidemia	400	(29)	198	(32)	0.174	186	(38)	162	(33)	0.110	0.102
Smoking	418	(30)	154	(25)	**0**.**013**	140	(28)	128	(26)	0.391	0.055
Cardiac family history	155	(11)	55	(9)	0.114	58	(12)	50	(10)	0.415	0.052
**Comorbidities**, **n** (%)											
Prior heart failure	264	(19)	216	(35)	**0**.**001**	167	(34)	185	(37)	0.232	0.076
Prior coronary artery disease	497	(36)	303	(49)	**0**.**001**	260	(52)	258	(52)	0.899	0.008
Prior myocardial infarction	317	(23)	154	(25)	0.350	169	(34)	133	(27)	**0**.**013**	0.158
Valvular heart disease	75	(5)	96	(15)	**0**.**001**	49	(10)	76	(15)	**0**.**010**	0.164
Prior ICD	129	(9)	109	(17)	**0**.**001**	61	(12)	90	(18)	**0**.**010**	0.164
Chronic kidney disease	551	(41)	344	(56)	**0**.**001**	244	(49)	266	(54)	0.162	0.089
Liver cirrhosis	18	(1)	9	(1)	0.788	9	(2)	5	(1)	0.282	0.068
COPD/asthma	114	(8)	77	(12)	**0**.**003**	54	(11)	63	(13)	0.376	0.056
Acute myocardial infarction	397	(29)	137	(22)	0.570	109	(22)	116	(23)	0.596	0.034
Cardiogenic shock	155	(11)	86	(14)	0.091	61	(12)	68	(14)	0.509	0.042
Non-ischemic cardiomyopathy	81	(6)	40	(6)	0.606	43	(10)	38	(9)	0.560	0.037
Stroke	28	(2)	33	(5)	**0**.**001**	11	(2)	27	(5)	**0**.**008**	0.169
Intracranial hemorrhage	8	(0.6)	6	(1)	0.334	2	(0.4)	3	(0.6)	1.000	0
**Cardiac therapies at index**, n (%)											
Cardiopulmonary resuscitation	526	(38)	218	(36)	0.217	162	(43)	163	(33)	0.946	0.004
In hospital	181	(13)	109	(18)	**0**.**008**	98	(20)	83	(17)	0.218	0.078
Out of hospital	347	(25)	109	(18)	**0**.**001**	64	(13)	80	(16)	0.149	0.092
External defibrillation	518	(38)	208	(34)	0.092	149	(30)	158	(32)	0.536	0.039
External cardioversion	47	(3)	58	(9)	**0**.**001**	15	(3)	52	(11)	**0**.**001**	0.210
**Coronary artery disease**, **n** (%)											
Coronary angiography, overall	924	(67)	350	(57)	**0**.**001**	355	(72)	303	(61)	**0**.**001**	0.210
Coronary artery disease, n (%)	660	(72)	261	(70)	0.126	260	(73)	208	(69)	**0**.**001**	0.210
None	264	(29)	89	(25)	0.166	95	(27)	75	(25)	0.677	
1-vessel	208	(23)	88	(25)		75	(21)	75	(25)		0.027
2-vessel	220	(24)	70	(20)		75	(21)	58	(19)		
3-vessel	232	(25)	103	(29)		110	(31)	95	(31)		
Chronic total occlusion	175	(19)	76	(22)	0.266	80	(23)	70	(23)	0.863	0.011
Presence of CABG	116	(13)	56	(16)	0.108	67	(19)	49	(16)	0.365	0.058
Intracoronary thrombus	79	(9)	24	(7)	0.325	15	(4)	23	(8)	0.065	0.117
**PCI**, n (%)	407	(44)	130	(37)	**0**.**026**	116	(33)	109	(36)	0.374	0.057
Target lesions											
Right coronary artery	153	(11)	60	(10)	0.351	50	(10)	52	(11)	0.834	0.013
Left main trunk	12	(0.9)	4	(0.6)	0.605	0	(0)	2	(0.4)	0.157	0.090
Left artery descending	210	(15)	60	(10)	**0**.**003**	51	(10)	52	(11)	0.917	0.007
Intermediate branch	8	(0.6)	2	(0.3)	0.452	6	(1)	1	(0.2)	0.058	0.121
Left circumflex	96	(7)	38	(6)	0.500	22	(4)	31	(6)	0.204	0.081
Bypass graft	10	(0.7)	2	(0.3)	0.283	3	(0.6)	2	(0.4)	0.654	0.029
**Left ventricular ejection function**, n (%)											
LVEF ≥55%	366	(34)	114	(22)	**0**.**001**	123	(25)	113	(23)	0.456	0.047
LVEF 54–35%	378	(36)	162	(32)	0.318	181	(36)	161	(32)	0.182	0.085
LVEF <35%	330	(31)	232	(46)	**0**.**001**	192	(39)	222	(45)	0.053	0.123
Not documented	302	—	192	—	—	—	—	—	—	—	
**Patients discharged at index**	1204	(88)	502	(81)	**0**.**001**	442	(89)	423	(85)	0.071	0.123
**Overall ICDs after discharge**, **n** (%)	573	(48)	289	(58)	**0**.**001**	269	(61)	253	(60)	0.753	0.021
ICD	514	(90)	249	(86)	0.123	243	(90)	216	(85)	0.082	0.119
CRT-D	36	(6)	32	(11)	**0**.**014**	24	(9)	31	(12)	0.215	0.084
s-ICD	23	(4)	8	(3)	0.354	2	(0.7)	6	(2)	0.130	0.103
**Medication at discharge**, n (%)											
Beta-blocker	939	(78)	415	(83)	**0**.**030**	383	(87)	356	(84)	0.299	0.071
Digitalis	92	(8)	117	(23)	**0**.**001**	47	(11)	101	(24)	**0**.**001**	0.225
Amiodaron	122	(10)	137	(27)	**0**.**001**	59	(13)	116	(27)	**0**.**001**	0.225
Vitamin K antagonist	168	(14)	262	(52)	**0**.**001**	91	(20)	226	(54)	**0**.**001**	0.225
Novel oral anticoagulant	56	(5)	119	(24)	**0**.**001**	32	(7)	106	(25)	**0**.**001**	0.225
Low molecular heparin	0	(0)	133	(27)	**0**.**001**	0	(0)	110	(26)	**0**.**001**	0.225

CABG, coronary artery bypass grafting; COPD, chronic obstructive pulmonary disease; CRT-D, cardiac resynchronisation therapy with defibrillator; ICD, implantable cardioverter- defibrillator; LVEF, left ventricular ejection fraction; PCI, percutaneous coronary intervention.

### Prognosis of AF and non-AF patients

The overall median long-term follow-up time was 3.8 years (IQR 257 days–7.6 years), whereas median survival time in AF-patients was 2.5 years (IQR 96 days–5.5 years) compared to a longer median of 4.7 years (IQR 1.2 years–8.3 years) in non-AF patients. The 2.5 years survival period derived from the affected AF patients was used for all outcome analyses.

**Figure 1:**
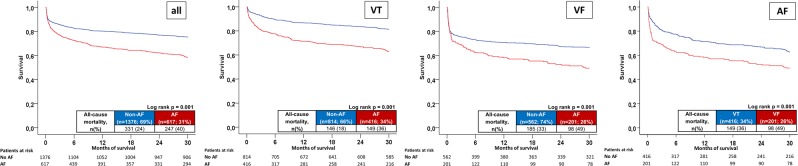
Primary endpoint: Long-term all-cause mortality at 2.5 years comparing AF with non-AF patients (first panel), stratified according to underlying ventricular tachyarrhythmias, VT (second panel), VF (third panel) and in AF patients respectively (fourth panel).

AF patients presenting with ventricular tachyarrhythmias were associated with a higher rate of the primary endpoint of long-term all-cause mortality at 2.5 years compared to non-AF patients (40% versus 24%; log-rank p = 0.001; HR = 1.825; 95% CI 1.548–2.153; p = 0.001; Fig. 1, first panel; Table 2 left columns). Increasing rates of all-cause mortality in AF patients were already observed for secondary endpoints at 30 days (17% versus 12%; HR = 1.467; 95% CI 1.146–1.878; p = 0.002), at index hospitalization (19% versus 13%; HR = 1.467; 95% CI 1.146–1.878; p = 0.002), and in patients surviving index hospitalization (21% versus 12%; HR = 1.467; 95% CI 1.146–1.878; p = 0.002) (Table 2 left columns).

**Table 2:** Primary and secondary endpoints. Entire cohort (left), matched cohort (right).

Characteristics	non-AF (n = 1,376; 69%)		AF (n = 617; 31%)		p value	non-AF (n = 496; 50%)		AF (n = 496; 50%)		p value
**Primary endpoint**, n (%)										
All cause-mortality, at 30 months	331	(24)	247	(40)	**0**.**001**	140	(27)	183	(37)	**0**.**004**
**Secondary endpoints**, n (%)										
All cause-mortality, at 30 days	160	(12)	104	(17)	**0**.**001**	47	(10)	66	(13)	0.058
In-hospital death at index	172	(13)	115	(19)	**0**.**001**	54	(11)	73	(15)	0.071
Death after discharge	159	(12)	132	(21)	**0**.**001**	86	(17)	110	(22)	0.056
**Follow up times**, n (%)										
Hospitalization total; days (median (IQR))	11 (6–19)		16 (8–29)		**0**.**001**	13 (8–22)		17 (9–29)		**0**.**001**
ICU time; days (median (IQR))	3 (3–8)		5 (0–11)		**0**.**001**	3 (0–8)		5 (0–12)		**0**.**001**
Follow-up; days (mean; median (range))	1847; 1700 (3–5106)		1241; 840 (443–3045)		**0**.**001**	1878; 1790 (513–2967)		1324; 911 (148–2129)		**0**.**001**

ICU, invasive care unit; IQR, interquartile range.

Increased mortality in patients with AF was observed also in VT (mortality rate 36% versus 18%; log rank p = 0.001; HR = 2.283; 95% CI 1.817–2.869; p = 0.001) (Fig. 1, second panel), and also in VF patients only (mortality rate 49% versus 33%; log rank p = 0.001; HR = 1.572; 95% CI 1.230–2.009; p = 0.001) (Fig. 1, third panel). Amongst AF patients, the presence of VF was associated with higher rates of the primary endpoint compared to VT (mortality rate 49% versus 36%; log rank p = 0.001; HR = 1.587; 95% CI 1.229–2.048; p = 0.001) (Fig. 1, fourth panel).

**Figure 2:**
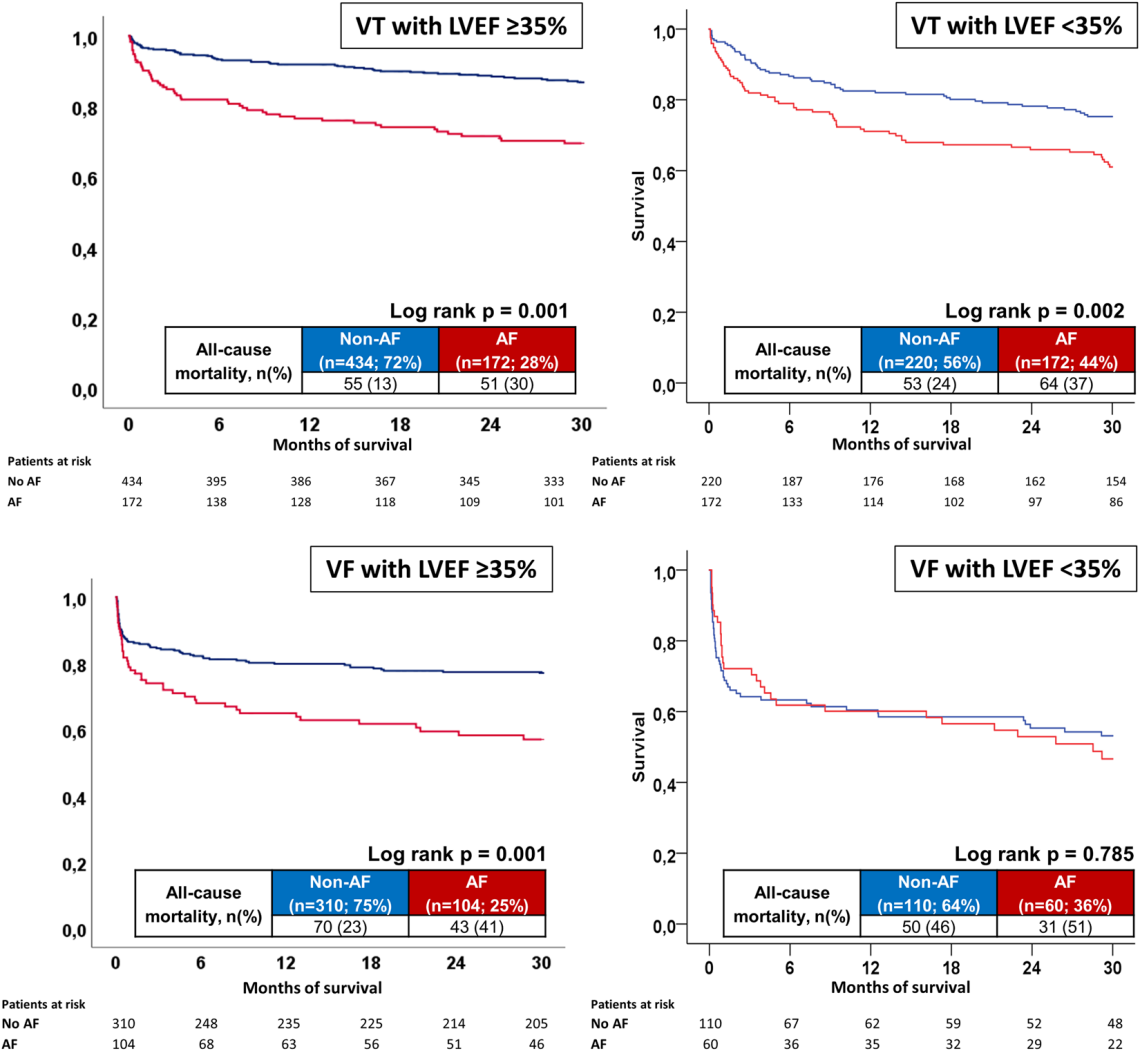
(**A**) VT and LVEF: Long-term all-cause mortality at 2.5 years between AF and non-AF patients presenting with VT and LVEF ≥35% (left) and <35% (right). (**B**) VF and LVEF: Long-term all-cause mortality at 2.5 years between AF and non-AF patients presenting with VF and LVEF ≥35% (left) and <35% (right).

**Figure 3:**
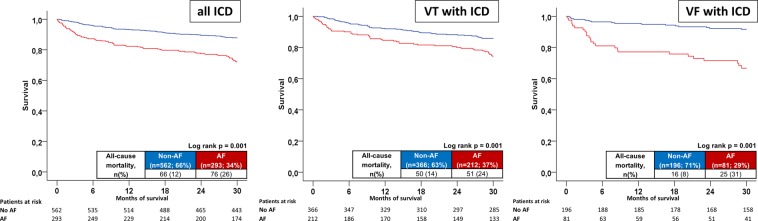
ICD patients: Long-term all-cause mortality at 2.5 years between AF and non-AF patients in patients with an ICD (left), stratified to VT (middle) and VF (right).

**Figure 4:**
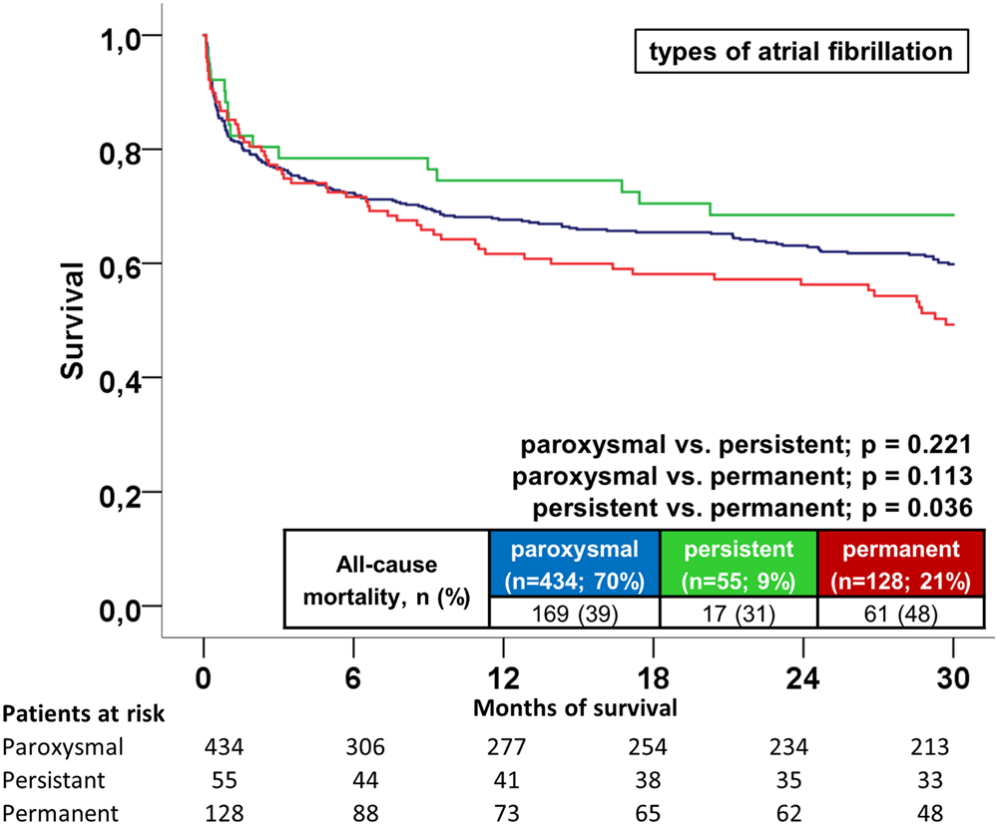
AF subtypes: Long-term all-cause mortality at 2.5 years according to types of AF.

Additionally, long-term all-cause mortality was significantly higher in AF patients irrespective of VT or VF in the presence of both LVEF ≥35% or <35% (p < 0.002) (Fig. 2A (VT patients) & B (VF patients), left panel: LVEF ≥35%; right panel: LVEF <35%). AF patients were still associated with a higher rate of long-term mortality in the presence of an ICD both within VT and VF patients (p = 0.001) (Fig. 3, left/middle/right panels). Patients with permanent AF revealed a higher rate of the primary endpoint compared to persistent AF patients (48% versus 31%, log-rank p = 0.036) (Fig. 4).

**Figure 5:**
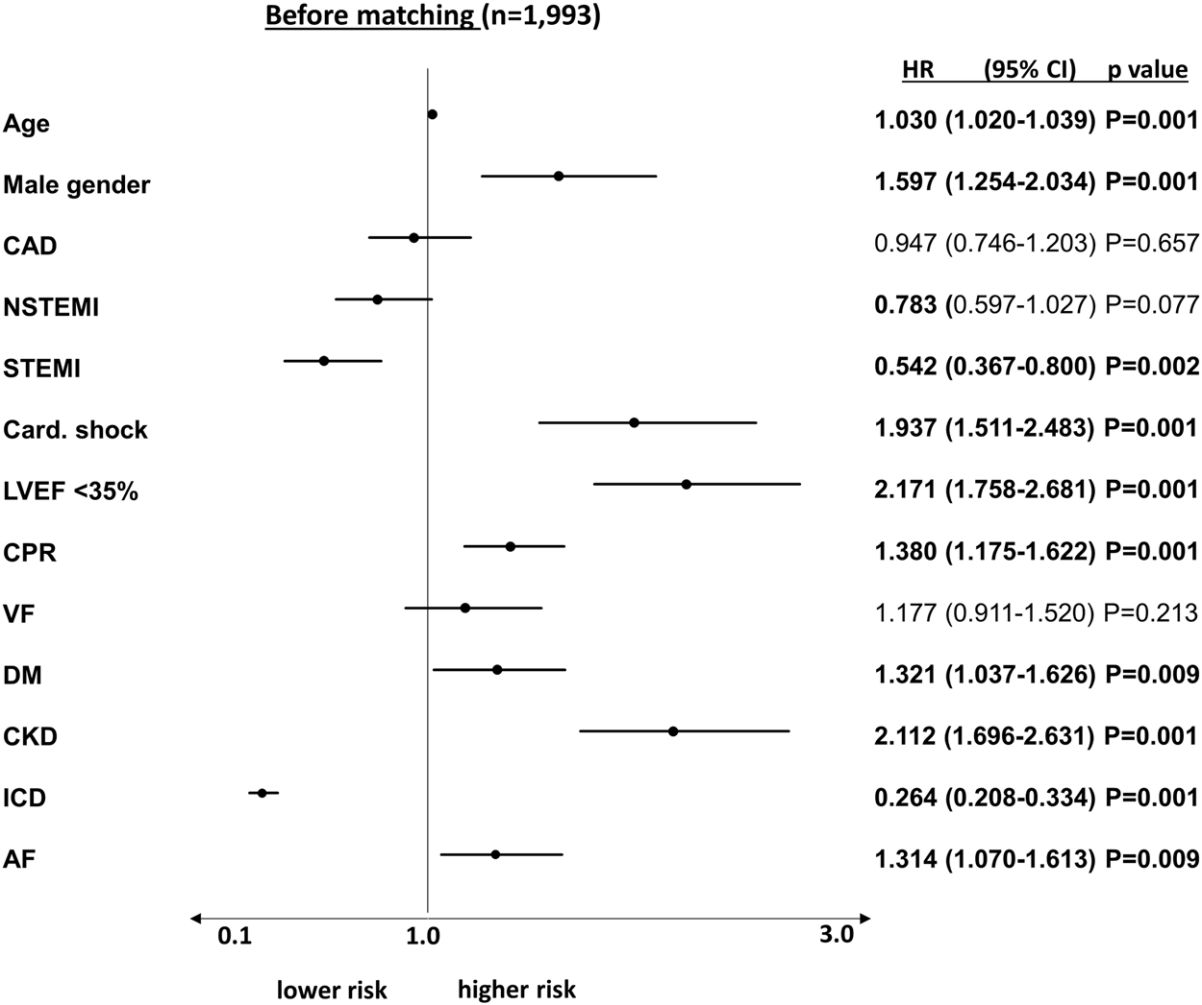
Multivariable Cox regression model: A history of AF was still associated with adverse long-term survival after adjusting for several prognosis-relevant factors.

In multivariable Cox regression models, AF patients presenting with ventricular tachyarrhythmias were at 1.3 times higher risk of the primary endpoint of long-term all-cause mortality at 2.5 years (HR 1.314; 95% CI 1.070–1.613; p = 0.009), besides age (HR 1.030), male gender (HR 1.597), diabetes (HR 1.321), STEMI (HR 0.542), chronic kidney disease (HR 2.112), LVEF <35% (HR 2.171), cardiogenic shock (HR 1.937), and CPR (HR 1.380) (Fig. 5).

### Matched study cohort

Since the present study includes consecutively patients presenting with ventricular tachyarrhythmias on admission some confounding might be present due to the heterogeneous comorbidities, which emphasize the real-life character of the present study population. Therefore, propensity score matching was performed for harmonization revealing similar baseline characteristics in each group (AF vs. non-AF each n = 496) except for minor differences in age, AF types, prior AMI, valvular heart disease, prior but not overall ICD, CAD, digitalis, amiodarone and anticoagulant therapies (Table 1, left columns).

**Figure 6:**
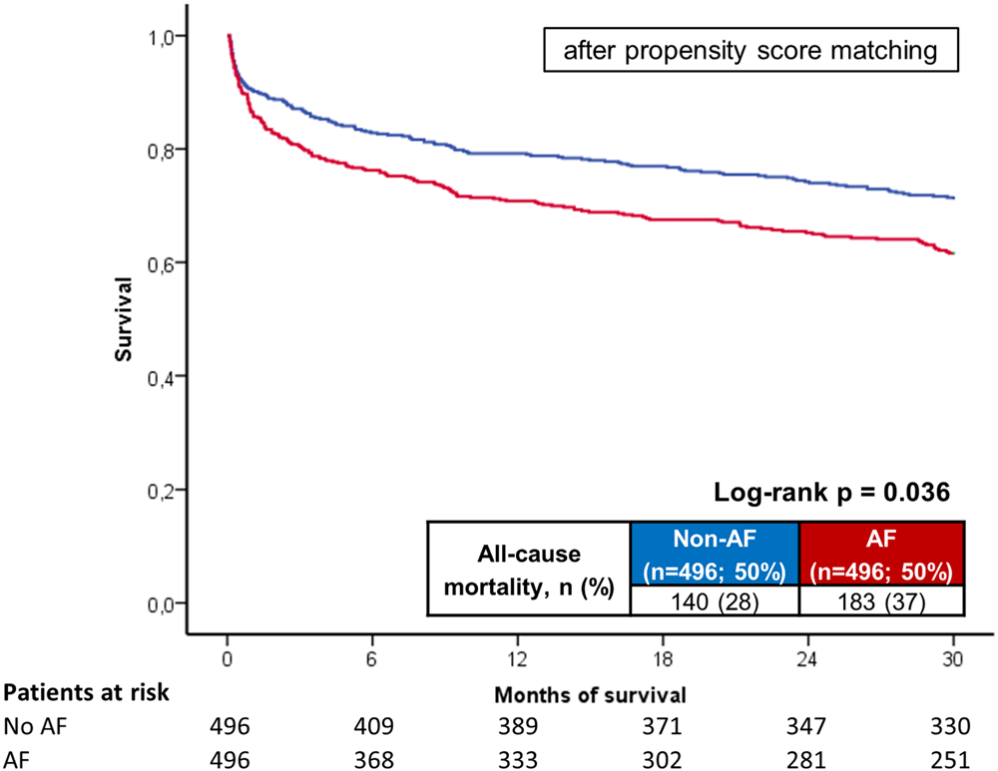
After propensity score matching: Long-term all-cause mortality at 2.5 years between AF and non-AF patients after propensity score matching.

Notably after propensity score matching, AF patients were still associated with increasing rates of the primary prognostic endpoint of long-term all-cause mortality at 2.5 years (long-term mortality rate 37% versus 28%, log rank p = 0.036; HR = 1.440; 95% CI 1.155–1.794; p = 0.001; Fig. 6; Table 2 right columns). Statistical trends were still observed for all predefined secondary endpoints even after propensity score matching (Table 2 right columns).

## Discussion

This real-world data suggests that a history of AF may be associated with an increased risk of long-term all-cause mortality in patients presenting with ventricular tachyarrhythmias. Mortality differences were already seen at 30 days, at index hospitalization and in patients surviving index hospitalization. They were irrespective of the presence of VT, VF, LV dysfunction and presence of an ICD. The prognostic disadvantage of AF was proven irrespective of LV dysfunction or presence of an ICD and was comparable to established risk factors such as cardiogenic shock, LVEF <35%, CPR, chronic kidney disease, diabetes and age, as well as after propensity-score matching.

This study consistently identifies the presence of AF as a robust predictor of adverse prognosis in patients presenting with ventricular tachyarrhythmias. On the one hand, AF patients are still endangered by an annual stroke rate of 1.5% impairing individual qualities of life due to varying physical disability. On the other hand, annual death rates of AF patients are estimated even higher (>3%) and are usually attributed to incident heart failure and SCD^2^. However, whether the presence of incident episodes of ventricular tachyarrhythmias in AF patients may impact prognosis has rarely been investigated. Most studies reported about higher rates of SCD or ventricular tachyarrhythmias in AF patients either in preselected ICD or population-based cohorts^2^.

The presence of AF has recently been shown to affect both clinically overt and subclinical alterations of ventricular structure and function. Potential pathologic mechanisms consist of the development of increasing ventricular rate, microvascular or endothelial dysfunction, systemic inflammation leading to consecutive impaired myocardial perfusion and heterogenous atrioventricular conduction^18^. In effect, increasing AF burden may be associated with impaired bidirectional atrio-ventricular supply-demand ischemia and progressive fibrosis sustaining adverse cardiac remodelling^18,19^. Beyond, atrial fibrillation may induce short-long-short sequences, premature ventricular complexes due to RR irregularity, QTc prolongation (>440 ms) and wide QRS (>130 ms) reflecting arrhythmogenic substrates for an increased vulnerability to ventricular tachyarrhythmias such as VT or VF during AF^20–25^.

Clinical studies showed evidence that the presence of AF was associated with increased rates of ventricular tachyarrhythmias and appropriate ICD shocks in ICD in the presence of LVEF <35%^26^. AF plus ventricular tachyarrhythmias may be detected simultaneously in about 8.9% of all detected ventricular tachyarrhythmias^27^. Presence of persistent AF was associated with progressive heart failure (LVEF <35%). Large-scaled population-based studies demonstrated a higher incidence of SCD at long-term follow-up in AF patients^28,29^. Furthermore, virtually all risk prediction scores for primary preventive ICD therapy found AF to be a strong risk factor in patients with LVEF <35%^30,31^. In contrast the adverse prognostic impact of AF was demonstrated in patients presenting with ventricular tachyarrhythmias even in the presence of LVEF >35%, as demonstrated in the present study.

The present results may evoke implications of how to assess patients with evidence of ventricular tachyarrhythmias and a history of AF. Risk stratification needs to be improved incorporating strategies to reduce the arrhythmogenic substrate both with respect to the AF burden and for ventricular tachyarrhythmias. This may in turn translate into a potential prognostic benefit for this specific subset of patients. Recent randomized controlled trials evaluating the impact of pulmonary vein isolation of AF demonstrated an improvement of quality of life and decrease of treatment-failure compared to optimal medical therapy^32–35^. Notably a prognostic benefit related to pulmonary vein isolation has only been demonstrated in patients with AF and concomitant heart failure with LVEF <35% with regard to lower rates of death from any cause or hospitalization for worsening heart failure^36^. The major finding of the present study showing that a history AF in patients presenting with ventricular tachyarrhythmias may negatively impact long-term prognosis reveals the urgent need for future prospective randomized controlled trials evaluating the prognostic impact of the following medical therapies in this sub-set of high-risk patients: (1) pulmonary vein isolation, (2) electrophysiological testing by exact electroanatomic mapping and consecutive VT ablation, (3) supply with an ICD, (4) the reduction of the ischemic burden by PCI of critical CAD and (5) a more aggressive therapy by antiarrhythmic drugs of class II or III. Until now, no clear recommendation can be given for these patients in daily routine care, unless these issues will not be evaluated as outlined.

Finally, the presence of AF needs to be accounted as another prognostic risk factor in patients with ventricular tachyarrhythmias. Classic risk factors such as LVEF <35% may reveal limited information, for instance in secondary preventive ICD recipients^37^, and since half of future SCD patients reveal a preserved ejection fraction irrespective of ischemic or non-ischemic origin^38,39^. Therefore the spectrum of potential risk factors for SCD has been extended towards myocardial scarring, reversible LV dysfunction, genetic determinants, ECG patterns and late gadolinium enhancement assessed by cardiac magnetic resonance imaging^37^. Except for VF patients with LVEF <35% reflecting progredient stages of heart failure, the presence of AF may sustain as another arrhythmic prognostic risk factor in patients presenting with ventricular tachyarrhythmias.

### Study limitations

This study has several limitations related to its study design as a registry-based, single-centre, retrospective and observational analysis. Therefore, the results of this study are at the most hypothesis-generating. However, performing a prospective study with adequate power and randomization of AF and non-AF patients is hard to investigate in future. In order to minimize potential selection bias retrospectively, propensity score matching was applied. Even thereafter some cofounding due to slight differences of age and further unmeasured variables in both groups, e.g. factors related to the out-of-hospital setting, may still be present. Therefore, a stricter matching was performed (calibre distance of 0.001) without further differences of age  and similar results. Mode of death was not documented mostly by registration offices. An independent clinical event committee was not applied, since all clinical data was documented reliably by individual cardiologists during routine clinical care being blinded to final data analyses. The additional prognostic impact of pulmonary vein isolation in AF patients was beyond the scope of the present study and needs to be evaluated in further prospective RCT. Whether AF itself is the cause or merely a symptom of underlying combination of disease processes in patients with ventricular tachyarrhythmias may not be drawn from the present results.

## Conclusions

AF is associated with increased secondary mortality in patients presenting with ventricular tachyarrhythmias.
